# Rectal shedding of monkeypox virus in a patient coinfected with *Chlamydia trachomatis* and *Neisseria gonorrhoeae*: a case report

**DOI:** 10.1186/s13256-023-03826-z

**Published:** 2023-03-06

**Authors:** Florian Desgranges, Emmanouil Glampedakis, Vanessa Christinet, Sara Encarnação, Cândida Fernandes, Gilbert Greub, Onya Opota, Matthias Cavassini

**Affiliations:** 1 Infectious Diseases Service, Lausanne University Hospital and University of Lausanne, Lausanne, Switzerland; 2 Cantonal Unit for Infection Control and Prevention, Public Health Service, Lausanne, Switzerland; 3 Sexual Health Clinic Checkpoint Vaud, Lausanne, Switzerland; 4 Emergency Department of Hospital de São José, Centro Hospitalar e Universitário de Lisboa Central, Lisbon, Portugal; 5 Dermatovenereology Department, Sexually Transmitted Diseases Clinic, Centro Hospitalar e Universitário de Lisboa Central, Lisbon, Portugal; 6 Institute of Microbiology, Lausanne University and University Hospital of Lausanne, Lausanne, Switzerland

**Keywords:** Monkeypox, Sexually transmitted infection, Proctitis, Shingles, Case report

## Abstract

**Background:**

Infection by the monkeypox virus classically causes a cutaneous rash that is preceded by fever and lymph node swelling, as well as other nonspecific systemic symptoms. A recent outbreak occurred and spread in Europe and other regions, especially among patients who declare themselves as men who have sex with men. Current reports have shown that cutaneous lesions may be limited to the anogenital area. We report on a case of proctitis caused by monkeypox virus, without visible typical lesions of this virus.

**Case presentation:**

A 29-year-old Caucasian male presented with a monkeypox virus proctitis that recurred after treatment for a documented *Neisseria gonorrhoeae* and *Chlamydia trachomatis* coinfection, likely acquired at the same time. The proctitis was preceded by fever and a swollen inguinal lymph node, and was associated with a hemorrhoid. The monkeypox virus polymerase chain reaction of a rectal swab documented high viral loads, although no typical lesion was visible. After resolution of the rectitis, the patient developed a single dermatome herpes zoster, despite the absence of usual risk factors. The patient evolved well without further specific treatment.

**Conclusion:**

This case shows that monkeypox virus can be responsible for proctitis, without any typical lesion, along with the important rectal shedding of the virus. It raises the concern of contagion during anal intercourse through body fluids and gives further credit that monkeypox virus can be a sexually transmitted infection. This should prompt routine rectal screening in patients with proctitis accompanied by fever and swollen lymph nodes, and in patients who have a history of unprotected receptive anal sex, even in presence of other sexually transmitted infections, and especially during a monkeypox virus outbreak. The potential link between monkeypox virus infection and shingles warrants further investigations.

## Introduction

Monkeypox virus (MPXV) is responsible for a recent global outbreak with more than 85,000 confirmed cases as of 30 January 2023, which seems to be mainly transmitted during sexual contact among people who declare themselves as men who have sex with men (MSM) [1]. Before this outbreak, the disease was described as a nonspecific flu-like disease, with fever and lymph node swellings, followed by a skin rash appearing 1–5 days later. The rash then evolves from macules to pustules for about 2–5 weeks [2]. Recent reports of the current epidemic describe a milder presentation and lesions may be limited to the genital area (3–6). Proctitis with a generalized maculopapular rash has been described in a recent case report (7). Our case is unique because the patient had documented MPXV proctitis with high viral load on rectal swab analysis, although typical lesions for MPXV infection were absent. Also, a similar presentation with coinfection by two sexually transmitted infections (STIs) has not been reported yet and is representative of the risk of missing the MPXV infection in such a scenario.

## Presentation

**Fig. 1:**
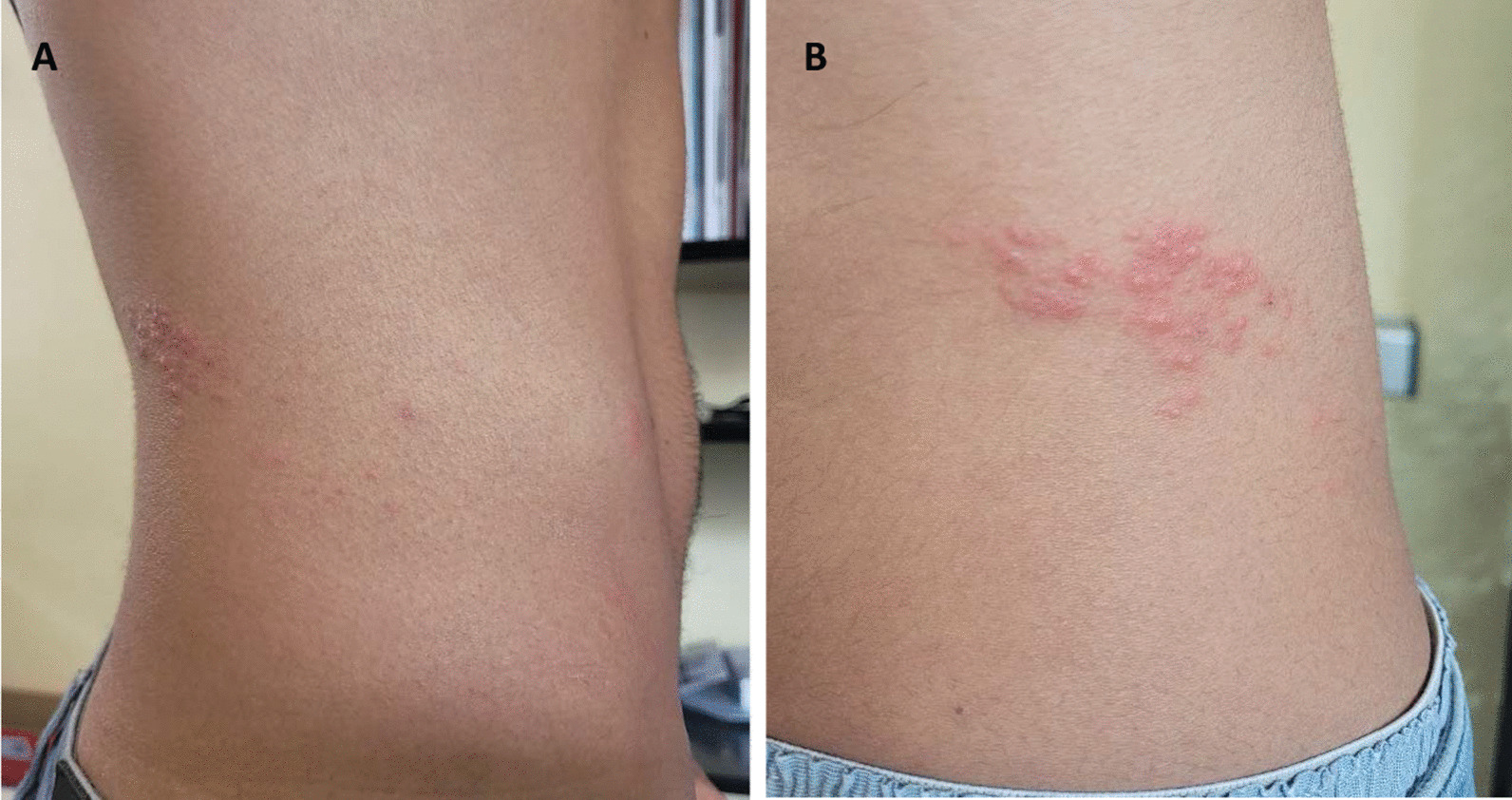
Shingles in the right T12 dermatome occurring 3 weeks after monkeypox virus infection. **A** Picture of the whole dermatome, **B** Zoom on the herpes zoster lesions

**Fig. 2:**
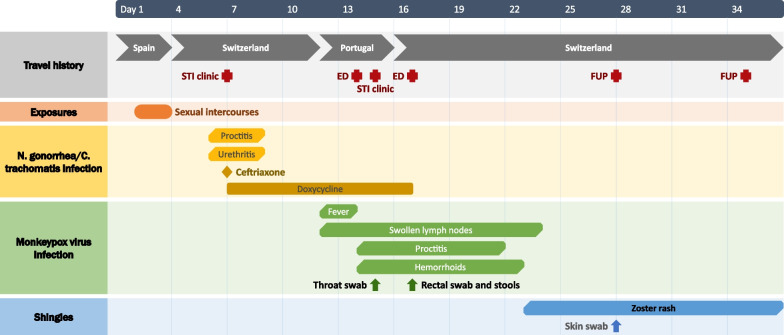
Timeline of the case report. Red crosses represent medical visits and arrows represent positive PCR samples. *ED* emergency department, *FUP* follow-up visit, *PCR* polymerase chain reaction, *STI* sexually transmitted infections

In mid-May 2022, a 29-year-old Caucasian male visited his sexual health clinic in Switzerland for acute urethral and anal purulent discharge and discomfort, where he was followed up for a human immunodeficiency virus (HIV) pre-exposure prophylaxis (PrEP). He had neither systemic nor oral symptoms. He reported having had several unprotected sexual intercourses with unknown male partners 4 days prior, during the Pride Parade of Maspalomas in the Canary Islands, Spain (5–15 May 2022). His past medical history is relevant for an enteric coinfection with *Shigella sonnei* and *Giardia lamblia* in late 2021. His regular STI follow-up was normal 3 months earlier. He was treated empirically for the current symptoms with one dose of intramuscular ceftriaxone (1 g) and a 7-day course of oral doxycycline (100 mg twice a day), in order to treat suspected *Neisseria gonorrhoeae* and *Chlamydia trachomatis* infections. Indeed, pooled oral, urethral, and anal swabs returned positive for both *N. gonorrhoeae* and *C. trachomatis* non-L serotype on polymerase chain reaction (PCR) tests. The symptoms resolved completely a couple of days later. Six days after the treatment start, the patient traveled to Portugal. On his day of arrival there, he developed high-grade fever (39 °C), as well as swollen and painful bilateral inguinal lymph nodes. On the next morning, he noticed two papular lesions on the left elbow fold that were slightly itchy. He visited an emergency department, where MPXV PCR was performed on a throat swab, as the two lesions were suggestive of an insect bite and no other skin rash was observed. In fact, the diagnosis was deemed unlikely, and no quarantine measure was established. On the next day, the anal pain and discharges reappeared but were less purulent than the week before. He visited an STD clinic in Portugal, where proctitis with hemorrhoids were diagnosed. Diosmine, paracetamol, and ibuprofen were prescribed, and doxycycline was prolonged for 7 more days. The patient then returned to Switzerland and, 6 days after the fever started, visited our emergency department for persisting proctitis. He reported that the fever resolved completely after 2 days and that the elbow skin lesions healed after 5 days, while the inguinal lymph nodes had decreased and become painless. He did not report any sexual relationship since his trip to the Canary Islands. Rectal examination revealed painful and swollen hemorrhoids, with neither perianal nor genital skin lesions, no anal discharge, nor any sign of prostatitis. Vital signs were normal, and neither vesicle, umbilicated lesion, nor crust were noted on the whole skin evaluation. A rectal swab ordered for *C. trachomatis* and *N. gonorrhoeae* returned negative, as well as screening tests for HIV, syphilis, and blood cultures. In addition, stools were analyzed for usual enteropathogens, including *Clostridioïdes difficile*, *Campylobacter* sp., *Salmonella* sp., *Shigella* sp., *Adenovirus*, *Rotavirus*, and *Norovirus*. This whole work-up returned negative and confirmed the cure of the previous STIs. On the same day, the patient also reported a notification from the Portuguese clinic for a positive throat swab for MPXV. Thus, MPXV PCR was also performed on the anal swab and stool sample and yielded positive results with cycle threshold values of 18.8 (corresponding to more than 1 million copies/milliliter) and 27.0, respectively. Following these results, public health authorities recommended home isolation until complete resolution of proctitis symptoms. Symptoms resolved spontaneously 10 days after the fever started. On the 12th day, a slightly painful papulovesicular rash appeared on the right side of his back and abdomen. The physical evaluation revealed typical grouped vesicles limited to the T12 dermatome with a slight hyperesthesia (Fig. 1). The PCR on the skin swab confirmed presence of varicella-zoster virus (VZV) deoxyribonucleic acid (DNA) in the lesions, while the MPXV PCR was negative, confirming the diagnosis of shingles. Lesions of shingles resolved after 2 weeks without further complication and no lesion suggestive of MPXV arose. The timeline of the case report is summarized in Fig. 2.

## Discussion

We report an unusual case of MPXV proctitis without typical skin lesions after a symptomatic sexually transmitted coinfection with *C. trachomatis* and *N. gonorrhoeae*. The first urethritis and proctitis symptoms that appeared 3 days after condomless sexual encounters can be attributed to the documented *C. trachomatis* and *N. gonorrhoeae* infections, as they completely resolved after adequate treatment. The second phase of the clinical picture with fever and inguinal adenopathies, followed by a second episode of proctitis, can be attributed to MPXV, with an incubation time of about 11 days. However, the patient had no typical MPXV lesions and the itchy skin lesions on the elbow that disappeared after 5 days were attributed to insect bites. Our experienced Portuguese colleagues decided to swab the throat as the patient had systemic symptoms (fever and lymph node swelling), similar to those recently reported [3]. The monkeypox-associated proctitis resolved spontaneously after 10 days.

This case is another example that MPXV infection can present as proctitis, as described in a recent report [7]. Interestingly, we also show that a documented MPXV infection does not always present with a rash and that there is a risk of missing the diagnosis, especially when other infections by pathogens that can cause proctitis are documented.

Our report supports the idea that MPXV can also be transmitted sexually, at least during anal intercourse. The current epidemic suggests that the infection can be acquired through genital or perianal lesions during sexual intercourse, similar to syphilis. Moreover, monkeypox is known to be present in body fluids [2], and this is supported by a recent case series, where MPXV DNA was documented by PCR in semen, feces, and rectal lesions [4]. Interestingly, we could observe high MPXV viral loads on rectal swabs and feces, even without macroscopically visible skin lesions. This raises the concern that typical skin lesions may not be visible at the time of patient presentation and strongly suggests that lesions may not be required for transmission of the disease during sexual intercourse. Thus, monkeypox should be part of the differential diagnosis for proctitis during a MPXV outbreak, especially when fever and swollen symptoms are present, and even if other agents of proctitis are detected.

Finally, the patient developed shingles 3 weeks after infection with MPXV, although he did not have the usual risk factors for reactivation, such as HIV, malignancy, or age [8]. A recent observational study in the Democratic Republic of Congo showed that MPXV and VZV can cause coinfections and we hypothesized that infection with MPXV may trigger VZV reactivation due to the immunomodulation induced during MPXV infection [9]. This, however, requires special attention from clinicians and further research.

The strength of the present case report is the distinct documentation of MPXV proctitis among two classical pathogens for proctitis (that is, *N. gonorrhoeae* and *C. trachomatis*). Also, we could demonstrate rectal shedding of high amounts of MPXV by a validated method, although skin lesions were completely absent at the time of presentation. We must acknowledge the limitation that we did not exclude internal rectal lesions by anuscopy, which would have been an unnecessary painful experience for the patient while he had a rectal inflammation. We cannot prove that the two skin lesions on the elbow were not due to the MPXV infection, as no swab was performed, but their description and rapid resolution suggest that they were not associated with this infection. Finally, we hypothesize that the patient acquired MPXV at the same time as *N. gonorrhoeae* and *C. trachomatis* because he reported no other sexual encounter after the first episode of proctitis, but this was impossible to prove as the first rectal swab was already discarded at the time of MPXV diagnosis.

## Conclusion

We report a case of monkeypox infection without typical skin lesions that was transmitted during sexual intercourse in parallel with two other STIs, and in whom proctitis was the main presenting symptom. We also showed that PCR appears to be more sensitive when performed on rectal swabs (compared with feces) and that it may be positive with very high viral load, even without visible skin lesions, suggesting a very high contagiousness already at that stage. Clinicians should be aware that MPXV can cause proctitis after anal intercourse and should include it in the differential diagnosis, especially during a MPXV outbreak and when symptoms are preceded by fever, lymph node swelling, and/or skin lesion(s), or if the symptoms persist after *C. trachomatis*/*N. gonorrhoeae* infections have been promptly identified and treated.

## Data Availability

Data sharing not applicable to this article as no datasets were generated or analysed during the current study.
